# Analysis of a machine learning–based risk stratification scheme for acute kidney injury in vancomycin

**DOI:** 10.3389/fphar.2022.1027230

**Published:** 2022-11-24

**Authors:** Fei Mu, Chen Cui, Meng Tang, Guiping Guo, Haiyue Zhang, Jie Ge, Yujia Bai, Jinyi Zhao, Shanshan Cao, Jingwen Wang, Yue Guan

**Affiliations:** ^1^ Department of Pharmacy, Xijing Hospital, Fourth Military Medical University, Xi’an, China; ^2^ Department of Health Statistics, School of Preventive Medicine, Fourth Military Medical University, Xi’an, China; ^3^ Department of Urology, Xijing Hospital, Fourth Military Medical University, Xi’an, China

**Keywords:** vancomycin, acute kidney injury, machine learning, stratification analysis, risk stratification

## Abstract

Vancomycin-associated acute kidney injury (AKI) continues to pose a major challenge to both patients and healthcare providers. The purpose of this study is to construct a machine learning framework for stratified predicting and interpreting vancomycin-associated AKI. Our study is a retrospective analysis of medical records of 724 patients who have received vancomycin therapy from 1 January 2015 through 30 September 2020. The basic clinical information, vancomycin dosage and days, comorbidities and medication, laboratory indicators of the patients were recorded. Machine learning algorithm of XGBoost was used to construct a series risk prediction model for vancomycin-associated AKI in different underlying diseases. The vast majority of sub-model performed best on the corresponding sub-dataset. Additionally, the aim of this study was to explain each model and to explore the influence of clinical variables on prediction. As the results of the analysis showed that in addition to the common indicators (serum creatinine and creatinine clearance rate), some other underappreciated indicators such as serum cystatin and cumulative days of vancomycin administration, weight and age, neutrophils and hemoglobin were the risk factors for cancer, diabetes mellitus, heptic insufficiency respectively. Stratified analysis of the comorbidities in patients with vancomycin-associated AKI further confirmed the necessity for different patient populations to be studied.

## Introduction

Acute kidney injury (AKI) is a common and severe renal disease that increases the risk of morbidity and mortality (Hoste et al., 2015). The basic strategy to address this disappointing situation is to identify drugs or factors that may cause or induce AKI in clinical practice to prevent subsequent AKI(Kan et al., 2022). The use of drugs is a modifiable risk factor for AKI, accounting for about 20%–40% of AKI in critically ill patients, and antibiotics are the key trigger of AKI in all drugs (Morales-Alvarez, 2020).

Vancomycin, a glycopeptide antibacterial agent, has tremendous potential to significantly reduce the incidence and severity of infections caused by methicillin-resistant *Staphylococcus aureus* (MRSA) and other Gram-positive beta-lactam-resistant bacteria over the past 50 years (Morales-Alvarez, 2020). In accordance with the guidelines for vancomycin therapy, the target AUC_0-24_/MIC of vancomycin was 400–600 mg*hour/L, and the steady-state trough concentration of vancomycin is 10–15 mg/L, those with severe infections maintain 10–20 mg/L (He et al., 2020). In recent guidelines from the American society of health-system pharmacists, trough-only monitoring with the target between 15 and 20 mg/L is no longer recommended in cases of serious infections caused by MRSA based on efficacy and nephrotoxicity data (Rybak et al., 2020). However, the treatment window for vancomycin was narrow and individual differences were considerable. Nephrotoxicity was the most serious adverse reaction to vancomycin, with 5%–43% of patients exhibiting vancomycin-associated AKI (van Hal et al., 2013). Another meta-analysis showed that the risk percentage of AKI attributable to vancomycin was 59% (Sinha Ray et al., 2016). In addition, the nephrotoxicity of vancomycin was usually closely related to the higher vancomycin daily dosage, longer duration of therapy, and elevated plasma concentrations of vancomycin (Fiorito et al., 2018) (Selby et al., 2019). Other factors, such as creatinine clearance (Ccr), blood urea (BU), alanine transaminase (ALT), aspartate transaminase (AST), and serum albumin (ALB), have also been reported to be associated with the risk of vancomycin-associated AKI(Li et al., 2018). However, for complex and variable real-world data, there is still a lack of effective identification of early warning factors. Different patient-specific explanations of underlying diseases, and the extent of impact of these factors on AKI, are still unknown.

Fortunately, machine learning may be able to provide a solution to this issue. It is widely acknowledged that machine learning is the basis of medical artificial intelligence, which has been used extensively in the field of medicine and healthcare (Alanazi et al., 2017). With the application of machine learning, models can be developed for early identification of disease risk, diagnosis of disease, recommendation of an appropriate dosing regimen, and visualization of data to interpret medical images (Deo, 2015; Hohmann, 2022). Compared with traditional methods, machine learning had the advantages of being more flexible, accurate, rapid and scalable in clinical application (Churpek et al., 2016; Ngiam and Khor, 2019). In previous studies, machine learning was used to predict AKI after cardiac surgery, in pediatric intensive care and cancer patients (Tseng et al., 2020; Dong et al., 2021; Scanlon et al., 2021). In addition, Kim et al. have developed a single-center vancomycin-associated AKI risk scoring system (Kim et al., 2022), which estimated of vancomycin area under the curve after vancomycin administration based on machine learning (Bououda et al., 2022). However, despite these advancements, all of these models were globally, which could not analyse and evaluate vancomycin-associated AKI in different underlying diseases specifically. These global models may ignore important and unique information specific to individuals with different underlying disease. Furthermore, the lack of interpretation studies for these models hinders their clinical application and basic research.

To achieve this, in this study, we proposed an AKI risk prediction framework for patients receiving vancomycin based on machine learning. Our framework focused on decision support and model interpretation for subtype patients with different underlying diseases. Firstly, based on XGBoost algorithm and electronic medical records (EMR) data, we built a series of machine learning models with good predictive performance using grid searching and cross-validation (Chen and Guestrin, 2016). Furthermore, the SHapley Additive exPlanation (SHAP) values were used to explain these prediction models from a global perspective for overcoming the shortcomings of machine learning models (Lundberg et al., 2018). The interpretative analysis revealed key clinical features of the AKI risk for patients with different underlying diseases. Finally, we conducted a stratified analysis from three underlying disease: cancer, diabetes mellitus, heptic insufficiency. The results have some implications for vancomycin-associated AKI clinical practice. Our study enables accurate predictions of the AKI risk in patients receiving vancomycin, the interpretation of key variables can be interpreted better and more accurately to support clinical decision making.

## Materials and methods

### Patient selection

The study was conducted at the Xijing Hospital of the Fourth Military Medical University, and a total of 724 patients were included in the study between 1 January 2015 to 30 September 2020. There were 638 patients in the control group and 86 patients in the AKI group. Our study was approved by the domestic ethics committee with the approval number KY20162010-2. This study is a retrospective, observational study design that does not require informed consent. It should be noted that all the collected data were de-identified and analyzed anonymously during the analysis process.

### Data collection

Through extensive literature review and consultation with health professionals, the following variables were selected for the analysis. Demographic data: age, gender, weight, hospitalization days and intensive care unit settings; Details on vancomycin treatment: vancomycin trough level, single dosage, dosing frequency, cumulative days, daily dosage and dosage; Concomitant disease: cancer (malignant tumours, neoplasms, leukemia, etc.), diabetes mellitus, hepatic insufficiency, pancreatitis, etc; Concomitant medications: human albumin, mannitol, loop diuretics (furosemide, torasemide and bumetanide), other antibacterial drugs (aminoglycosides, amphotericin B, piperacillin-tazobactam, meropenem, imipenem-cilastatin), other nephrotoxic medications (cyclosporine, tacrolimus, platinum compounds, dobutamine, dopamine, epinephrine, isoproterenol, norepinephrine and vasopressin); Laboratory indicators: white blood cell count (WBC), absolute neutrophil value (NEUT#), creatinine (CRE), creatinine clearance (Ccr), blood urea (BU), alanine transaminase (ALT), aspartate transaminase (AST), and serum albumin (ALB), etc.; Finally, we got 51 variables in total (including derived variables). All variables were described and classified in detail in the supplementary section (Supplementary Tables S1, S2).

When the vancomycin dose was changed in the middle of administration, the trough value measured before the dose change was used if it was after 3 days from the start of administration, and if not, the trough value measured after 3 days from the dose change was used (Izumisawa et al., 2020). Daily dosage (mg/day) was calculated by Single dosage (mg)*Dosing frequency (/day); Dosage (mg/day/kg) was calculated by Daily dosage (mg/day)/Weight (/kg). Drugs administered with vancomycin for >2 days were considered to be concomitant medications.

### Inclusion criteria and exclusion criteria

Study participants were patients who were hospitalized at Xijing Hospital of Fourth Military Medical University from 1 January 2015, through 30 September 2020, and who received vancomycin treatment for ≥48 h and whose vancomycin trough concentrations were measured after the third day after administration. The following patients were excluded: 1) if they were under the age of 18; 2) if they already suffered from renal diseases at the start of vancomycin, because renal diseases interfered the judgment of the main outcome-AKI. And renal diseases were diagnosed by clinicians according to International Classification of Diseases-10, including uremia or dialysis, kidney transplant, kidney failure, chronic kidney disease and acute renal injury; 3) if estimated creatinine clearance (Ccr) ≤45 ml/min, which has been calculated using Cock Croft-Gault formula based on baseline creatinine (CRE), weight, age and gender; 4) if patients already had a rising CRE prior to or just before starting vancomycin; 5) if vancomycin was administered orally rather than intravenously; 6) if vital information, such as vancomycin trough concentration, baseline and subsequent CRE, had been omitted.

**FIGURE 1:**
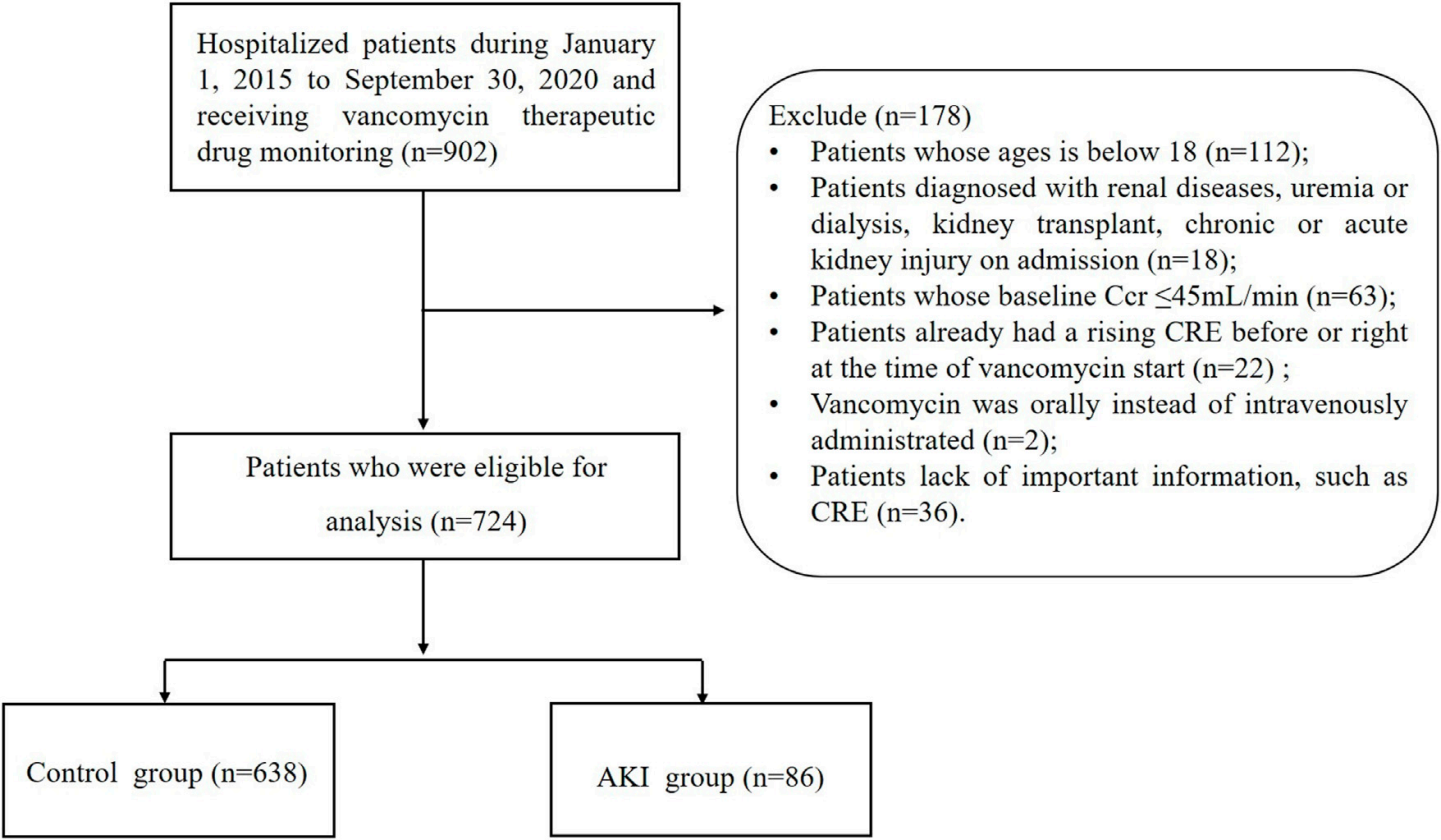
Flowing chart of population enrollment.

Patients in this study were divided into two groups based on their AKI status: the control group and the AKI group. It was established in the derivation cohort and independently validated in the validation cohort. Additionally, data were collected by the same investigators, typically using the same predictors and outcome definitions and measurements. An overview of the selection process for patients can be found in Figure 1.

### Criteria of vancomycin-associated AKI

All the blood samples were collected by nurse according to medical orders and measured by the laboratory in 1 day. In order to obtain vancomycin trough concentrations, samples were collected half an hour prior to administration of the drug. The main outcome was incidence of AKI during the period of vancomycin treatment, which was defined as an increase in serum creatinine of ≥0.5 mg/dl (44.2 μmol/L) or a 50% increase from baseline on two or more consecutive measurements (Rybak et al., 2009; Aljefri et al., 2019).

### Preprocessing and imputation of clinical variables

The clinical variables could be divided into numerical and categorical variables based on their clinical significance. For longitudinal variables containing multiple measurement, only the most recent measurement before blood concentration monitoring was kept. Then, the categorical variables were converted into one-hot vectors. A detailed description and classification of all variables can be found in the supplementary section (Supplementary Table S2). Variables which had more than 20% missing values were deleted. Multivariate imputation by chained equations (MICE) were used to impute missing values while loss rates of variables less than 20%. Finally, we got 34 variables in total.

### Model algorithm

XGBoost (eXtreme Gradient Boosting) is a scalable tree boosting system, which is versatile, and efficient gradient enhancement framework developed by Chen et al. (Chen and Guestrin, 2016). To create boosted, DT-type models, XGBoost employs the ensemble of weak DT-type models. In addition to handling sparse data, XGBoost can be used to solve a lot of data science problems efficiently and accurately. In many machine learning challenges, it has been widely used by data scientists to obtain state-of-the-art results. The equations were as follow:

$$L\left(\emptyset \right)=\overset{n}{\underset{i}{\sum }}l\left({\hat{y}}_{i},y_{i}\right)+\overset{k}{\underset{j}{\sum }}\Omega \left(f_{j}\right)$$ (1)

Here, Loss function l represents the difference between the prediction ${\hat{y}}_{i}$ and the target $y_{i}$ . The Ω penalizes the complexity of the model. Here, all XGBoost models were implemented by using XGBoost (version 1.5.1). All code was implemented using Python 3.7.9.

### Evaluation metrics

To evaluate the performance of XGBoost models, the averaged performances of accuracy (ACC), area under the receiver operator characteristics curve (AUROC), and area under the precision recall curve (AUPRC) for each model were calculated and compared.

### Interpretation algorithm

SHAP were used to interpret the results from the models of XGBoost. SHAP is a framework for interpretation of model prediction based on Shapley values, which is a sum of individual features influencing the model. In order to quantify the relative importance of each parameter, Shapley values were aggregated as follow:

$$g\left(z^{\prime }\right)=\emptyset_{0}+\overset{M}{\underset{i=1}{\sum }}\emptyset_{i}z_{i}^{\prime }$$ (2)

Where $z^{\prime }\in {\left\{0,1\right\}}^{M}$ , M is the number of simplified input feature, and $\emptyset_{i}\in R$ . $\emptyset_{0}$ and $\emptyset_{i}$ is the interpretation model constant and the predicted mean value of all training samples respectively.

### Statistical analysis

In this study, a series of kernel density estimation (KDE) plots are used to analysis observations. In a KDE plot, observations are visually displayed and smoothed with a Gaussian kernel, resulting in a continuous density estimate. Violin plots shown the broken line indicates are upper quartile, median and lower quartile. Statistical analysis was performed using two independent-sample t-tests, with significance defined as a *p*-value of less than 0.05. All statistical analyses were performed using Scipy 1.7.2.

## Results

### General information

A total of 724 patients were eventually enrolled in this study, and the baseline demographic characteristics of the study patients are shown in Table 1. There were 62.7% (n = 454) males and 30.7% (n = 222) admitted to the ICU. The median age and weight of the study population were 51.0 years (IQR 39.0–63.0 years) and 70.0 kg (IQR 55.0–70.0 kg), respectively. As indicated by the median serum creatinine level and creatinine clearance, respectively, they were 75 μmol/L (IQR 63.0–92.0 μmol/L) and 88.0 ml/min (IQR 64.7–110.7 ml/min). The most common complication was cancer (n = 188, 26.0%), followed by hypertension (n = 150, 20.7%) and heptic insufficiency (n = 107, 14.8%), and diabetes mellitus (n = 68, 9.4%). The incidence of vancomycin-associated AKI was 11.88% (86 of 724 patients).

**TABLE 1:** Characteristics of patients at baseline and clinical outcomes.

Categories	Variables	Total (n = 724)
Basic information	Age (years) [median (IQR)]	51.0 (39.0–63.0)
	Male [No. (%)]	454 (62.7%)
	Weight (kg) [median (IQR)]	70.0 (55.0–70.0)
	Hosp (days) [median (IQR)]	33 (16.0–42.0)
	ICU [No. (%)]	222 (30.7%)
	Surgery [No. (%)]	342 (47.2%)
Vancomycin administration	Trough level (mg/L) [median (IQR)]	8.7 (4.8–14.7)
	Dosing frequency (/day) [median (IQR)]	2.0 (2.0–3.0)
	Single dosage (mg) [median (IQR)]	1,000 (500–1,000)
	Daily dosage (mg/day) [median (IQR)]	2000 (1,500–2000)
	Dosage (mg/kg/day) [median (IQR)]	28.6 (25.9–36.4)
	Cumulative days (days) [median (IQR)]	6.0 (4.0–10.0)
Concomitant diseases	Cancer [No. (%)]	188 (26.0%)
	Diabetes mellitus [No. (%)]	68 (9.4%)
	Hypertension [No. (%)]	150 (20.7%)
	Heptic insufficiency [No. (%)]	107 (14.8%)
	Pancreatitis [No. (%)]	23 (3.2%)
	Shock [No. (%)]	33 (4.6%)
	Heart Failure [No. (%)]	8 (1.1%)
Concomitant medications	Human albumin [No. (%)]	382 (52.8%)
	Mannitol [No. (%)]	243 (33.6%)
	Loop diuretics [No. (%)]	420 (58.0%)
	Other antibacterial drugs [No. (%)]	383 (52.9%)
	Other nephrotoxic medications [No. (%)]	45 (6.21%)
Laboratory Indicators	WBC(×10^9/L) [median (IQR)]	8.3 (5.2–12.4)
	NEUT# (×10^9/L) [median (IQR)]	6.1 (3.2–10.0)
	NEUT% [median (IQR)]	0.8 (0.6–0.8)
	LYMPH# (×10^9/L) [median (IQR)]	1.2 (0.7–1.7)
	MONO# (×10^9/L) [median (IQR)]	0.5 (0.3–0.8)
	RBC (×10^12/L) [median (IQR)]	3.4 (2.9–3.9)
	PLT (×10^9/L) [median (IQR)]	209.5 (103.8–292.0)
	HGB (g/L) [median (IQR)]	102.0 (86.0–116.0)
	ALT (IU/L) [median (IQR)]	33.0 (18.0–58.3)
	AST (IU/L) [median (IQR)]	27.0 (17.0–44.2)
	ALP(IU/L) [median (IQR)]	86.0 (66.0–118.0)
	GGT (IU/L) [median (IQR)]	50.5 (29.0–95.1)
	TBIL (μmol/L) [median (IQR)]	11.3 (7.7–18.2)
	CRE (μmol/L) [median (IQR)]	75.0 (63.0–92.0)
	BU (mmol/L) [median (IQR)]	5.4 (3.7–8.4)
	BU/ CRE [median (IQR)]	18.0 (12.1–25.4)
	CCr (ml/min) [median (IQR)]	88.0 (64.7–110.7)
	CysC (mg/L) [median (IQR)]	1.0 (0.8–1.3)
	ALB (g/L) [median (IQR)]	32.8 (30.1–35.4)
	FIB (g/L) [median (IQR)]	4.0 (3.2–4.6)
	TT(s) [median (IQR)]	16.9 (16.2–17.8)
	D-Di (mg/L FEU) [median (IQR)]	3.7 (2.0–5.0)
	PCT (ng/ml) [median (IQR)]	1.5 (0.3–1.9)
	IL-6 (pg/ml)) [median (IQR)]	147.6 (32.5–152.7)
	CRP (mg/L) [median (IQR)]	31.2 (11.4–88.3)
	SAA (mg/L) [median (IQR)]	97.2 (28.5–206.5)
	ESR (mm/h) [median (IQR)]	47.0 (27.2–73.5)

**FIGURE 2:**
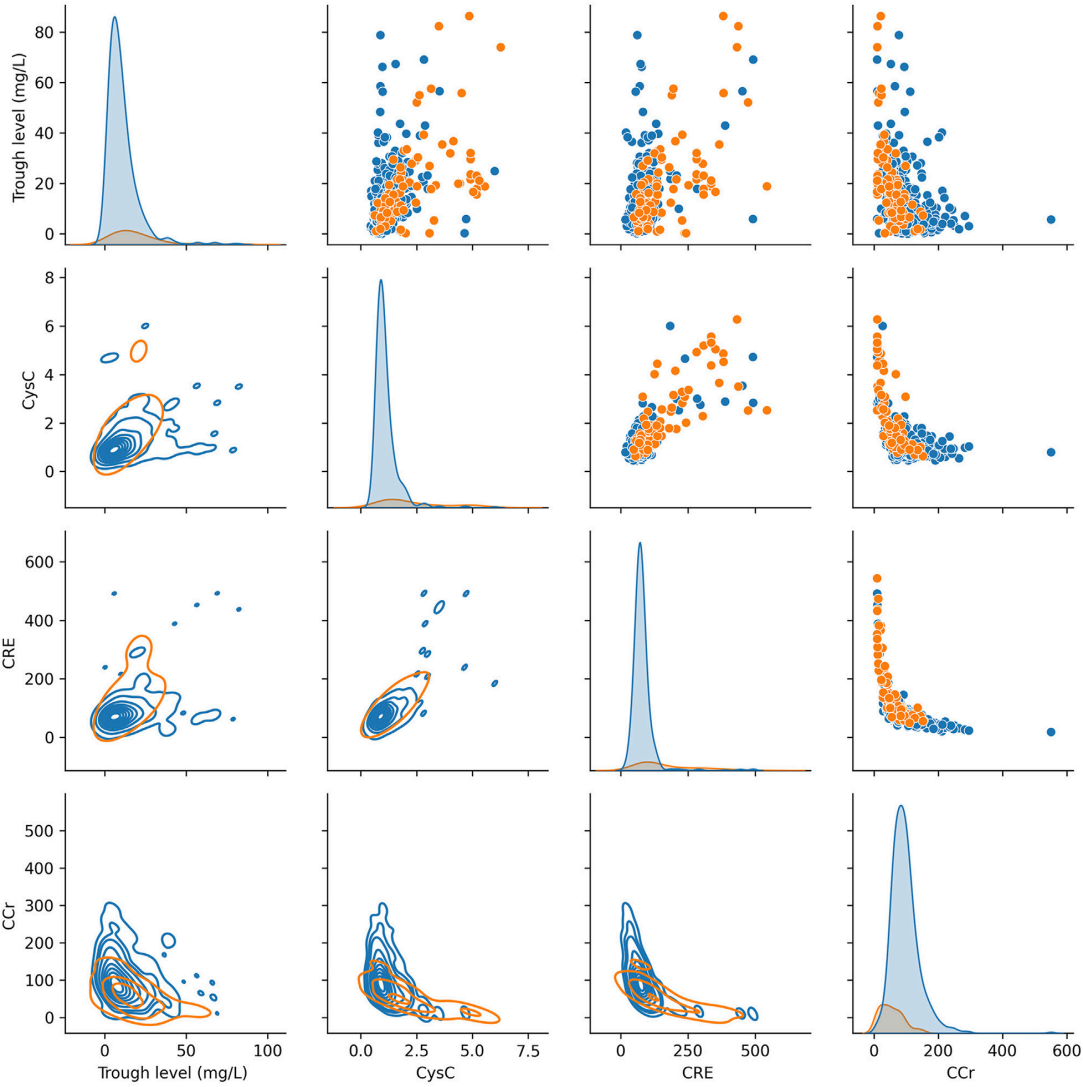
KDE plots of key indicators for vancomycin-associated AKI (on the diagonal). Scatter plot analysis (above the diagonal). KDE plots of conditional distributions with 2D Gaussian (under the diagonal).

In addition, we used the KDE to display bivariate distributions of the key indicators reported in the previous literatures (CRE, Ccr, CysC and trough level). As shown in Figure 2, it was found that a single indicator was difficult to define the risk of vancomycin-associated AKT. Therefore, it is necessary to construct a machine learning model for predicting more risk factors of vancomycin-associated AKI.

### Stratification analysis

**FIGURE 3:**
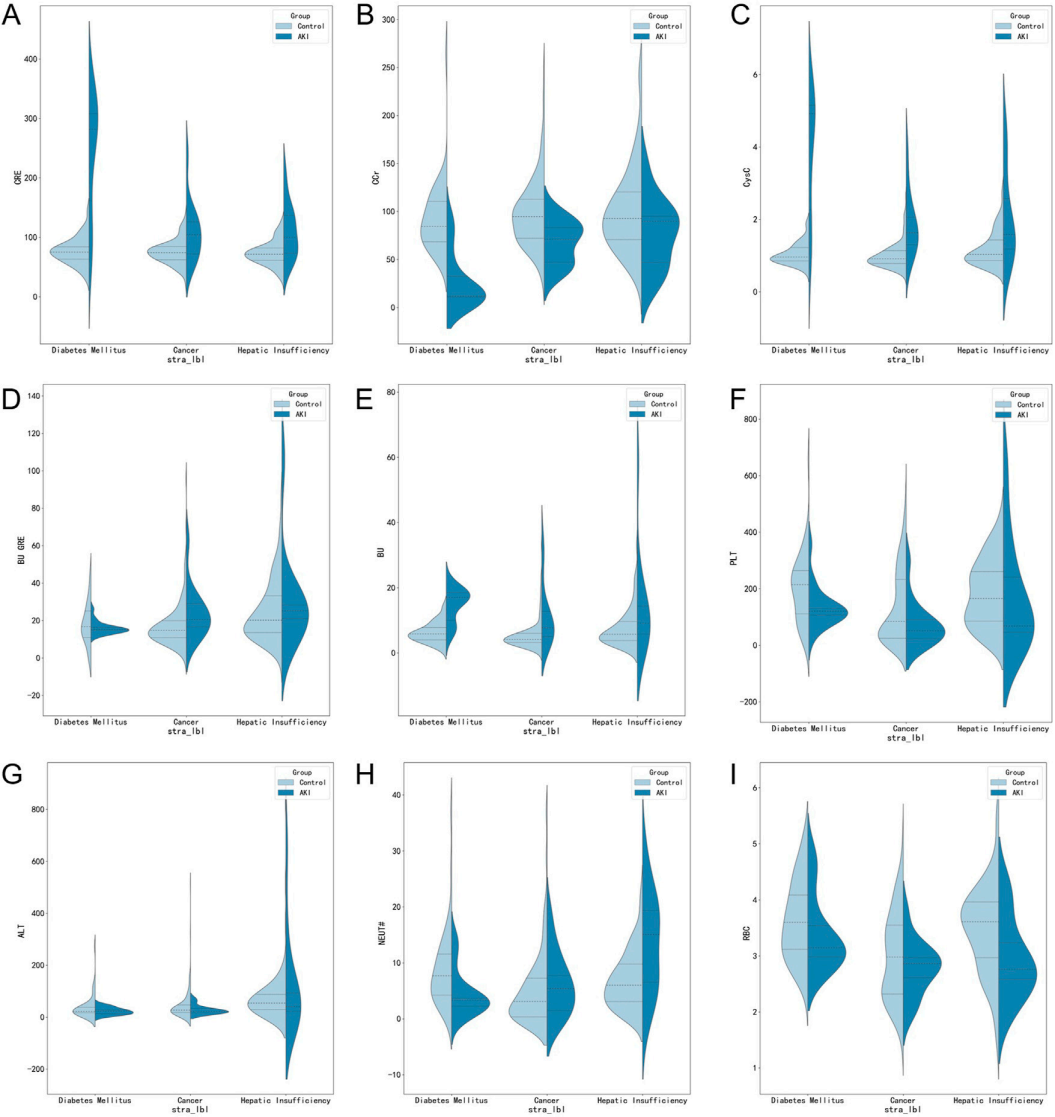
Violin plot for stratified analysis in different underlying diseases.

There was highly heterogeneous response to vancomycin-associated AKI, and the severity of the presentation varies by subpopulation. Therefore, when the discussion was stratified by comorbidities (cancer, n = 188; diabetes mellitus, n = 68; heptic insufficiency, n = 107), we found significant differences between groups as well as within groups in the following aspects, as illustrated in Figure 3: On the one hand, patients with cancer, diabetes mellitus, and heptic insufficiency showed significant differences in CRE, Ccr, CysC, and PLT, and these differences were statistically significant (*p* < 0.05 or *p* < 0.01). On the other hand, an intra-group analysis showed that the levels of CRE, Ccr, CycC, BU, PLT and NEUT were different in diabetic patients with AKI (*p* < 0.05 or *p* < 0.01), whereas for cancer patients, the levels of CRE, Ccr, CycC and BU were statistically different (*p* < 0.01), while NEUT# and RBC were significantly different in patients with heptic insufficiency (*p* < 0.05 or *p* < 0.01).

### Model optimization and performance

**FIGURE 4:**
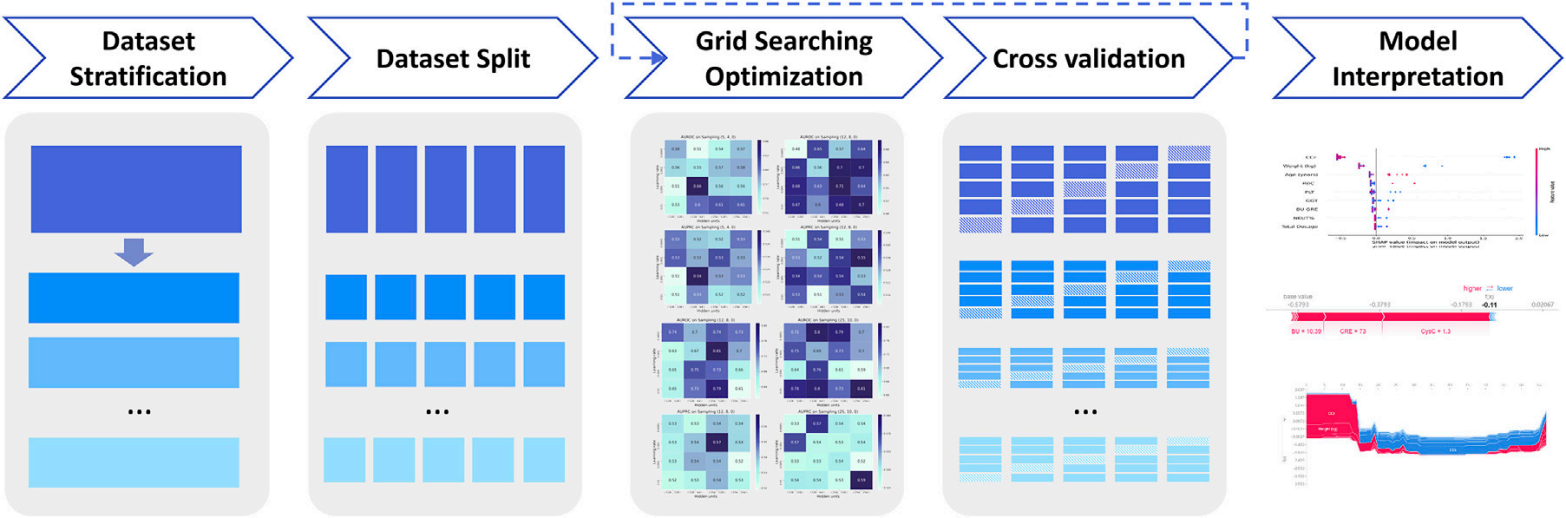
Overall flowchart of the study.

To overcome the high heterogeneity of vancomycin-associated AKI in patients with different underlying diseases, we built respective sub-models with different underlying diseases. The workflow of XGBoost machine learning algorithm is shown in Figure 4 **.** In addition to the three diseases mentioned above, two new classifications of ICU patients and non-initial patients have been added to the sub-model. Patients who have been dose-adjusted during treatment and whose vancomycin concentration was not at its initial trough are referred to as non-initial patients.

The global data was divided according to underlying diseases of patients, and then the global data set and the underlying disease data set were further divided into five parts, respectively. One of the five sets was selected as test set, the rest four sets were selected as training set. To optimize each XGBoost models, different hyperparameters were explored through grid search, including the maximum depth, the number of estimators and learning rate. We considered the maximum depth with 2, 4, 8,10, 16, 20 and 32, the number of estimators with 2, 4, 8, 10, 16, 20, and 32, the learning rate with 0.01, 0.02, 0.05, 0.1, 0.2, 0.25, 0.3 and 0.5. The best hyperparameters were selected according to the mean performance based on cross validation.

In addition, the ACC, AUROC and AUPRC of mean performance of 5-fold cross validation were displayed in Table 2. The results shown that the vast majority of sub-model performed best on the corresponding sub-dataset.

**TABLE 2:** The ACC, AUROC, and AUPRC of global model and sub-model.

Model	Global ACC		Cancer ACC		Diabetes mellitus ACC	Heptic insufficiency ACC	ICU ACC		Non-initial ACC
Global	0.912 ± 0.014		0.928 ± 0.036		0.907 ± 0.042	0.864 ± 0.048	0.901 ± 0.013		0.955 ± 0.026
Cancer	0.884 ± 0.019		0.936 ± 0.043		0.875 ± 0.015	0.876 ± 0.057	0.848 ± 0.018		0.886 ± 0.059
Diabetes mellitus	0.869 ± 0.026		0.905 ± 0.047		0.915 ± 0.046	0.869 ± 0.018	0.871 ± 0.041		0.896 ± 0.046
Heptic insufficiency	0.866 ± 0.027		0.885 ± 0.062		0.739 ± 0.155	0.882 ± 0.047	0.858 ± 0.033		0.87 ± 0.046
ICU	0.899 ± 0.018		0.916 ± 0.046		0.79 ± 0.125	0.909 ± 0.026	0.905 ± 0.03		0.91 ± 0.03
Non-Initial	0.906 ± 0.016		0.893 ± 0.049		0.903 ± 0.042	0.917 ± 0.023	0.889 ± 0.03		0.957 ± 0.012

Model	Global AUROC			Cancer AUROC	Diabetes mellitus AUROC	Heptic insufficiency AUROC		ICU AUROC	Non-Initial AUROC
Global	0.879 ± 0.039			0.676 ± 0.294	0.848 ± 0.092	0.788 ± 0.142		0.87 ± 0.093	0.927 ± 0.044
Cancer	0.776 ± 0.051			0.794 ± 0.192	0.845 ± 0.093	0.653 ± 0.158		0.808 ± 0.068	0.86 ± 0.108
Diabetes mellitus	0.678 ± 0.091			0.678 ± 0.259	0.857 ± 0.103	0.527 ± 0.164		0.723 ± 0.118	0.847 ± 0.114
Heptic insufficiency	0.696 ± 0.065			0.669 ± 0.201	0.818 ± 0.223	0.866 ± 0.128		0.833 ± 0.038	0.718 ± 0.204
ICU	0.773 ± 0.048			0.577 ± 0.149	0.845 ± 0.069	0.781 ± 0.197		0.877 ± 0.078	0.917 ± 0.042
Non-Initial	0.757 ± 0.039			0.574 ± 0.177	0.854 ± 0.073	0.616 ± 0.226		0.808 ± 0.046	0.939 ± 0.04

Model	Global AUPRC	Cancer AUPRC			Diabetes mellitus AUPRC	Heptic insufficiency AUPRC	ICU AUPRC		Non-Initial AUPRC
Global	0.829 ± 0.014	0.885 ± 0.098			0.596 ± 0.144	0.842 ± 0.038	0.774 ± 0.053		0.781 ± 0.091
Cancer	0.817 ± 0.016	0.895 ± 0.066			0.648 ± 0.092	0.855 ± 0.054	0.784 ± 0.06		0.823 ± 0.079
Diabetes mellitus	0.815 ± 0.032	0.891 ± 0.093			0.677 ± 0.159	0.856 ± 0.057	0.73 ± 0.026		0.821 ± 0.088
Heptic insufficiency	0.825 ± 0.022	0.902 ± 0.075			0.668 ± 0.077	0.867 ± 0.024	0.78 ± 0.05		0.84 ± 0.066
ICU	0.797 ± 0.017	0.901 ± 0.082			0.614 ± 0.094	0.851 ± 0.061	0.785 ± 0.058		0.802 ± 0.083
Non-Initial	0.809 ± 0.005	0.894 ± 0.053			0.67 ± 0.108	0.863 ± 0.039	0.782 ± 0.046		0.854 ± 0.062

### Model interpretation: Importance of clinical variables for different underlying diseases

Although the vast majority of sub-model can achieve good predictions performance, the lacking of interpretation limits the application in clinical practice and further differentiation analysis. In order to facilitate interpretation of each sub-models, the Shaply values have been introduced, which can indicate the positive or negative relationship of clinical variable with prediction.

**FIGURE 5:**
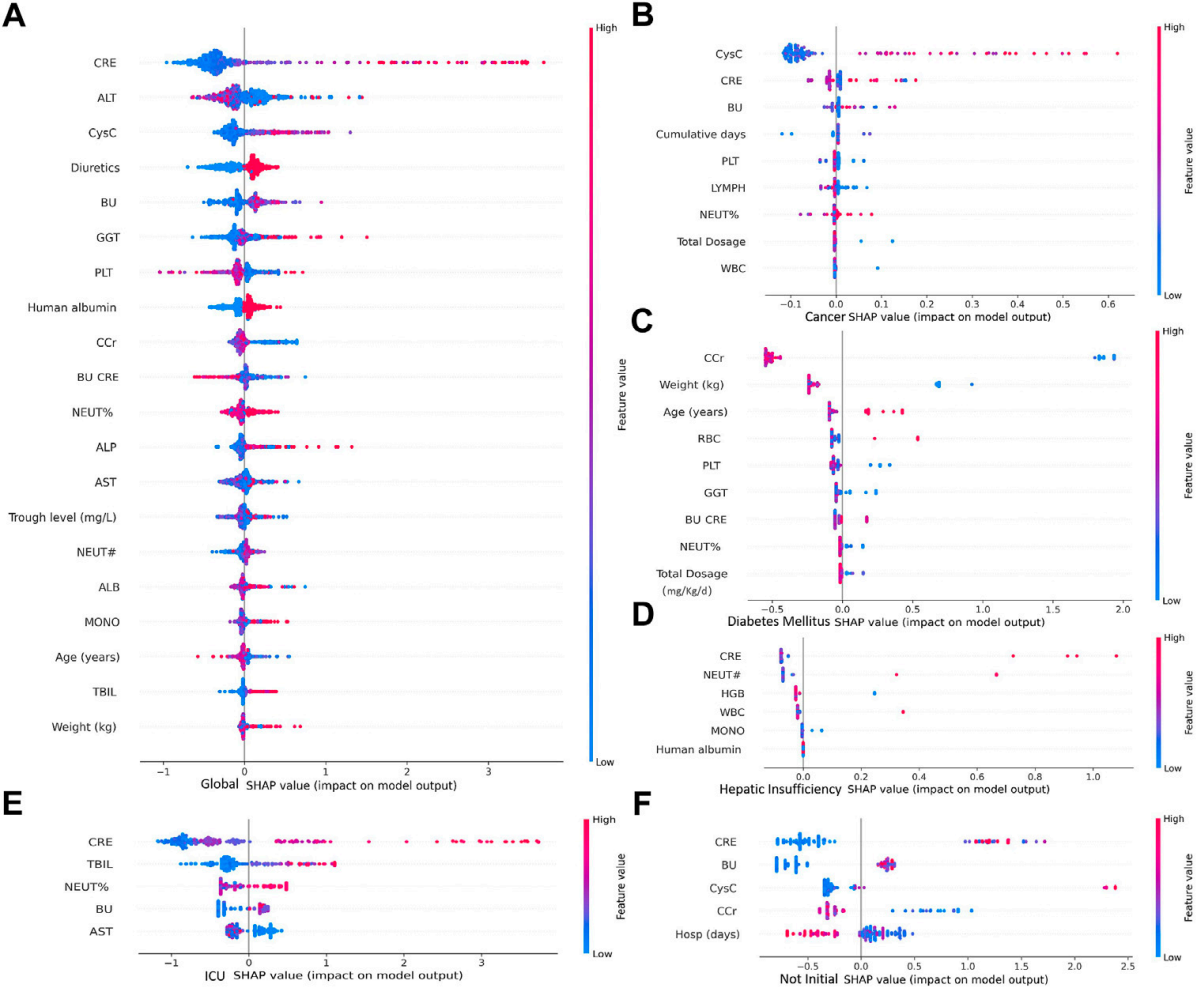
Importance of clinical variables for different underlying diseases. In a bee swarm plot, each point corresponding to a sample of data set. The position of each point on the horizontal axis indicated the effect of that feature on the model prediction, and the color of a point reflected the eigenvalue of the case. Variables were ranked in descending order of importance in terms of their impact on the model predictions, with the variable on top being the most important.

According to the importance and impacts of variables on model prediction, a bee swarm plot was formed for each feature. As shown in Figure 5, a series bee swarm plots were listed in their order of importance. In global model, we found that patients with high CRE (red) had a higher risk of developing AKI than patients with low CRE (blue). Similarly, patients with higher CysC, lower Ccr, and lower urea nitrogen/creatinine ratio (BU/CRE) had a higher risk of developing AKI. Moreover, patients who used diuretic and human albumin solution had a higher risk of mortality than those who not used. Liver function indicators (eg, ALT, GGT, ALP, and AST) also showed different risk of AKI, Figure 5A shows the specific trends. Among the sub-model of disease classification, CysC and Ccr had the highest impact on model output in cancer and diabetes patients, respectively. In heptic insufficiency, ICU and non-initial patients, CRE had the highest impact on model output. While several laboratory features (eg, BU, PLT, NEUT, WBC, GGT and HGB), and concomitant medications (eg, human albumin) were also highly ranked. The result is shown in Figures 5B–F. Notably, in ICU patients, high TBIL may be a high risk factor for AKI.

**FIGURE 6:**
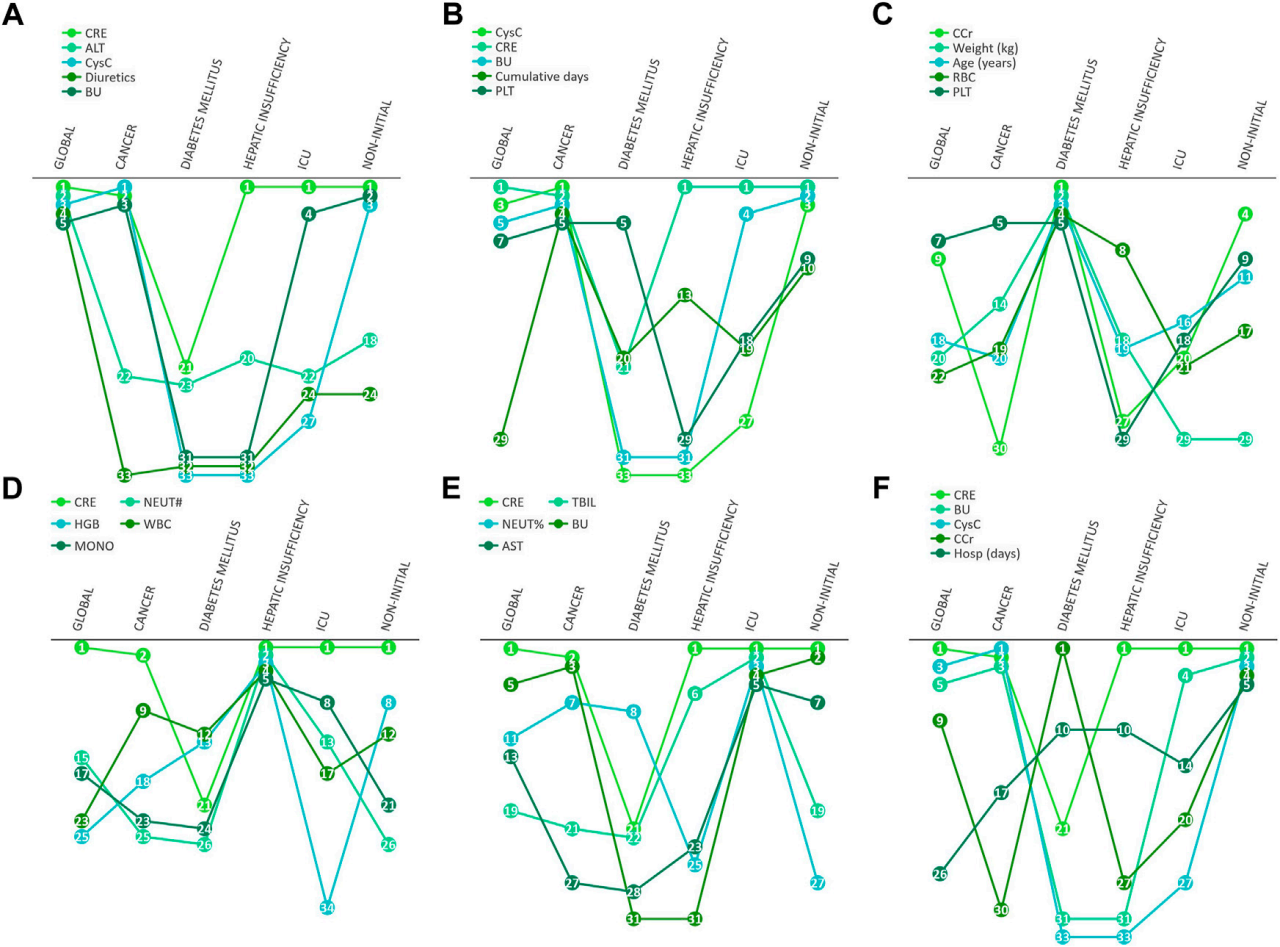
Differential manifestations analysis. The number in cycle means the rank number of clinical variables in different underlying diseases.

In further differential analysis, we found that ALT was a risk factor for diagnosing AKI in the vancomycin patient population, but this indicator was not as important in cancer or diabetes patients (Figure 6A). In contrast, for patients with cancer, CysC and the cumulative of days of vancomycin administration were specific and important indicators (Figure 6B). In addition, age and weight were key factors in determining whether AKI occurs for diabetic patients (Figure 6C). In patients with hepatic insufficiency, their NEUT#, HGB, WBC and MONO# need to be concerned (Figure 6D). In addition, it is worth mentioning that PLT was a class of clinical indicators that had been previously usually overlooked but are quite important in most patient populations. For patients in intensive care unit, CRE, TBIL, NEUT% and BU were specific and important indicators (Figure 6E). In patients with non-initial patients trough, their CRE, BU, CysC and CCr need more attention (Figure 6F).

### Model interpretation: Personal and population explanations stratification analysis for different underlying diseases

Regarding local interpretation, the SHAP force plots can provide a visualized explanation for personal and population stratified prediction, which can assist clinicians to analyze individual patient so that personalized interventions can be targeted.

**FIGURE 7:**
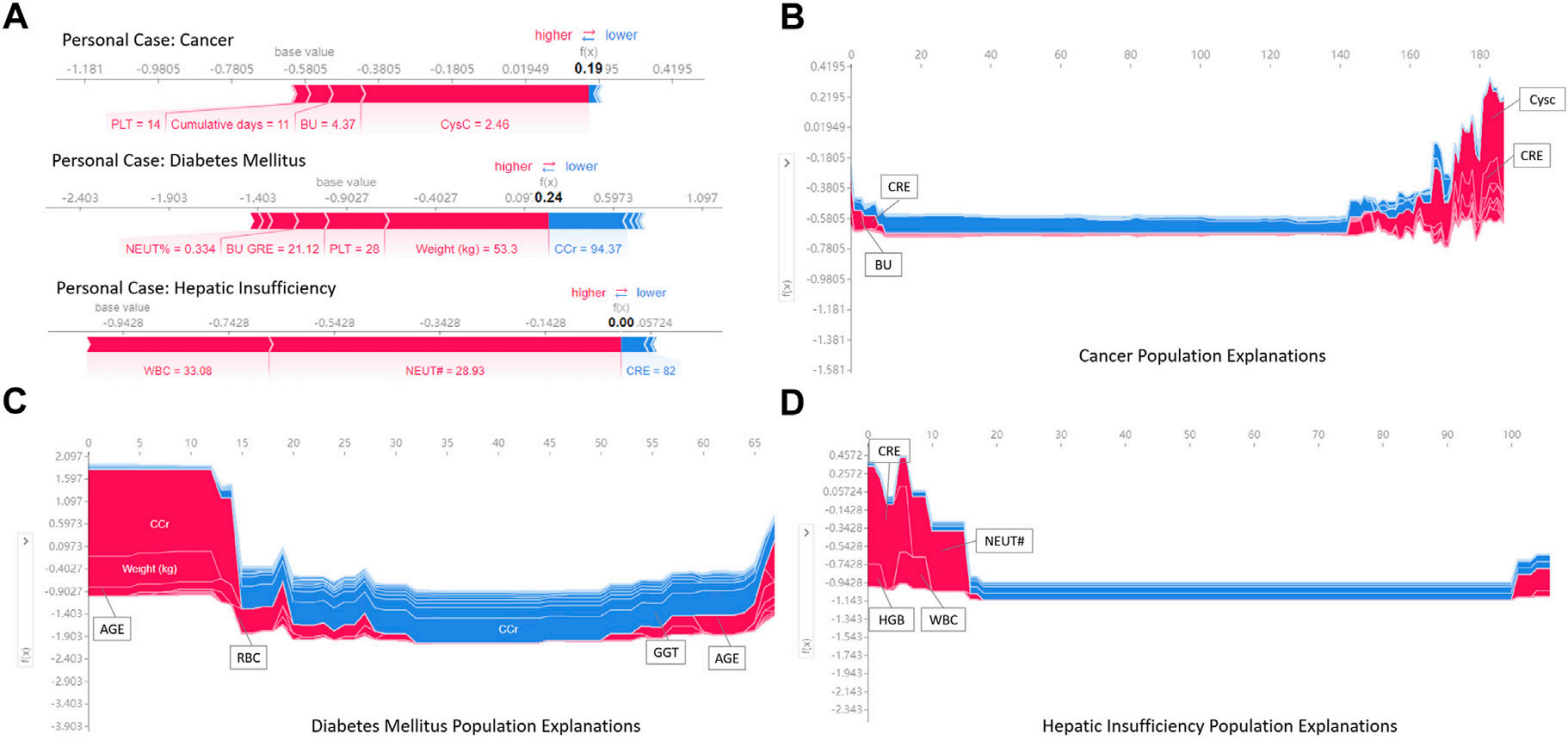
Examples of using SHAP values to explain the prediction results. The features pushing higher the predicted probability of AKI are shown in red, and those pushing the prediction lower are shown in blue. Additionally, the length of the bars corresponds to the contribution of each factor.

Figure 7A illustrates the results of analysis to individualize treatment using our model to predict the risk of vancomycin-associated AKI in patients with cancer, diabetes and heptic insufficiency, respectively. The key parameters and their the risk of developing AKI was quantified. For example, in a heptic insufficiency patient, the fact that the WBC and NEUT# were high, pushed the predicted severity score higher even if the CRE was in normal range. In the case of diabetes mellitus patients, most parameters being outside the normal range (such as a patient had a high weight and PLT, and high NEUT% and BU/CRE), the probability of vancomycin-associated AKI was increased.

Figures 7B–D is a series of population explanation analysis. A collection of all individual analysis samples (like Figure 7A) is listed, then clustered and sorted by sample similarity. These are used to analyze the characteristics of the patient population using vancomycin. The visualization showed the key risk factors in the risk determination of AKI for the samples in different underlying diseases groups. Patients with cancer were judged to be at high risk of AKI usually due to high CysC, CRE and BU (Figure 7B). Diabetic mellitus patients were at high risk of AKI due to older, high CCr and RBC (Figure 7C). Patients with hepatic insufficiency were at high risk of developing AKI due to higher CRE, NEUT#, WBC, and HGB (Figure 7D). Additionally, all effects in the model only describe the model’s behavior and are not causal in the real world.

## Discussion

Vancomycin-associated AKI remains a major challenge for patients and clinicians. Therefore, it is of great significance to predict the risk factors of AKI using vancomycin in critically ill patients. In this study, we assessed the risk factors of 724 patients with monitoring of vancomycin plasma concentration, included 86 vancomycin-associated AKI and 638 control group. The incidence of vancomycin-associated AKI in this study was 11.88%, which was lower than the incidence reported in the literature without monitoring blood concentration (Gaggl et al., 2020), suggesting that monitoring vancomycin may reduce renal injury. In this study, we established a series of machine learning models focused on different underlying diseases for AKI incidence risk of vancomycin. Compared to global model, the vast majority of sub-model achieved the best performance in ACC, AUROC, and AUPRC on the corresponding sub-dataset. Furthermore, the SHAP values were introduced to assist in determining whether there is an association between clinical variables and risk of AKI, as well as to evaluate the importance of clinical variables in predicting AKI risk. Finally, analyses stratified based on the three underlying diseases (cancer, diabetes mellitus and hepatic insufficiency) provided a visual interpretation of each prediction. The experimental results showed that the prediction and interpretation framework had good prediction and interpretation performance and was expected to provide effective support for clinical decision making.

Clinical risk estimation models are commonly trained as global models. This study found that global models were significantly associated with patient heterogeneity and did not work equitably well across subpopulations. Therefore, the underlying disease-based prediction model were established and stably applied to the prediction of vancomycin-associated AKI. Patients with cancer have been reported to have a higher renal clearance, which could result in insufficient exposure to vancomycin when the drug was administered at conventional dosage rates (Zhang and Wang, 2020). Consequently, there may be an increase in resistance and a failure to respond to treatment, which can result in a higher rate of infection-related morbidity and mortality. Despite the fact that systemic inflammatory response syndrome and malignancy may both be risk factors for the development of vancomycin-associated AKI, this phenomenon and its causal consequences have rarely been considered in most studies (Song and Wu, 2022). These results suggest that early monitoring of vancomycin concentration in these patients may be critical for maintaining desired effects without the occurrence of side effects. (Nakayama et al., 2019; Izumisawa et al., 2020). Other studies have shown that diabetes mellitus and heptic insufficiency are independent risk factors for vancomycin-associated AKI (Chambers et al., 2020; Wang et al., 2021). It is well known that diabetes mellitus can cause kidney disease. Studies have shown that the survival rate of patients with diabetic nephropathy was much lower than that of patients without diabetic nephropathy. It is therefore possible that diabetic patients who take nephrotoxic drugs may be at greater risk of developing nephrotoxicity (Chen et al., 2017). Therefore, this study can better predict the occurrence of AKI by establishing different comorbidity sub-model, which was also verified by the results of model establishment.

Additionally, the most obvious finding to emerge from the analysis above was that CRE, ALT, CysC, BU, GGT, PLT, Ccr and BU/CRE and other indicators were risk factors for the development of vancomycin-associated AKI, which was consistent with the results of previous studies (Frazee et al., 2017; Griffin et al., 2019). In our global model, loop diuretics and human albumin were also identified as risk factors for vancomycin-associated AKI. For example, human albumin is excreted by the kidney during kidney injury, so supplementing with human albumin can aggravate kidney injury, resulting in further damage to already damaged kidneys, a worsened glomerular basement membrane charge barrier and mechanical barrier, as well as an increase in protein leakage, which may be a risk factor for vancomycin-associated AKI (Kimura et al., 2021). In addition, combined use of diuretics and vancomycin, both nephrotoxic agents, has been reported to result in renal damage (Wu et al., 2014). Therefore, the accumulation of these biomarkers can be used as an important indicator of decreased renal clearance and was of great significance for the prediction of AKI. More importantly, for vancomycin users with different comorbidities, the frequency and risk factors of AKI are different (Figure 3). As for cancer patients, CysC is clinically ranked as the first predictor of AKI, several studies suggest that cancer patients should measure their CysC level before vancomycin administration, which can effectively ensure safe and effective dose (Zhang and Wang, 2020). In addition, age and weight were also critical for the prediction of AKI risk in patients with diabetes, which was consistent with previous reports. As for patients with hepatic insufficiency, this model suggests that, neutrophils, hemoglobin, white blood cell and monocytes all need to be concerned. To sum up, stratified analysis of comorbidities (cancer, diabetes mellitus, hepatic insufficiency) in patients with vancomycin-associated AKI further confirms the need for studies targeting different patient populations.

This retrospective study still had several limitations. Firstly, this was a single-center study, which limits the generalizability of our findings. Secondly, we restricted our risk estimates for AKI to the duration of hospitalization, and previous studies have shown that important predictors may vary between time windows, and the baseline important factors that acute physiology and chronic health evaluation II scores, sequential organ failure assessment scores, could not be evaluated. In future works, our model should be futher optimized in multi-centric real world clinical data and other public clinical database. Thirdly, with the emergence of more evidence-based evidence, the latest consensus guideline advocated clinicians should monitor the efficacy and nephrotoxicity of vancomycin by calculating the AUC/MIC ratio rather than trough-only monitoring, but in this study we were unable to complete the collection of this potential factor due to the absence of more monitoring sites for vancomycin concentration data. Last but not least, all conclusion only described the behavior of models and are not causality in the real world, and expanding the sample population in the future may be one of the effective measures to reduce such biases. In conclusion, we developed a global and disease-stratified methodological risk prediction model to quantified the risk of vancomycin-associated AKI. This prediction model and individualized interpretation framework can help clinicians make informed decisions to adjust vancomycin administration to reduce the risk of AKI.

## Conclusion

In summary, machine learning algorithm of XGBoost was used to construct a series risk prediction model for vancomycin-associated AKI in different underlying diseases. The vast majority of sub-model achieved the best performance in ACC, AUROC, and AUPRC on the corresponding sub-dataset. Additionally, stratified analysis of the comorbidities (cancer, diabetes mellitus, heptic insufficiency) in patients with vancomycin-associated AKI further confirmed the necessity for different patient populations to be studied.

## Data Availability

The original contributions presented in the study are included in the article/Supplementary Material, further inquiries can be directed to the corresponding authors.

## Supplementary material

Supplementary Table S1 The detail list of concomitant medications.

Supplementary Table S2 The full information of clinical variables collected in this study.

## Abbreviations

AKI, acute kidney injury; ALT, alanine transaminase; AST, aspartate transaminase; ALB, serum albumin; ACC, averaged performances of accuracy; AUROC, area under the receiver operator characteristics curve; AUPRC, area under the precision recall curve; ALP, a lkaline phosphatase; BU, blood urea; Ccr, creatinine clearance; CRE, creatinine; CysC, cystatin-C; EMR, electronic medical records; GGT, gamma-glutamyl transpeptidase; HGB, hemoglobin; KDE, kernel density estimation; LYMPH#, lymphocyte count; MONO#, monocyte count; MRSA, methicillin-resistant *Staphylococcus aureus*; MICE, multivariate imputation by chained equations; NEUT#, neutrophil count; NEUT%, neutrophilic granulocyte percentage; PLT, platelet count; RBC, red blood cell count; SHAP, SHapley Additive exPlanation; TBIL, total bilirubin; WBC, white blood cells.
