# Supplementary material for: Analysis of a machine learning–based risk stratification scheme for acute kidney injury in vancomycin
Source: Front Pharmacol. 2022 Nov 24;13:1027230. doi: 10.3389/fphar.2022.1027230 (PMC9730034; doi:10.3389/fphar.2022.1027230)
Supplement: Supplementary file 2 [file Table1.docx]

**TABLE S1** | The detail list of concomitant medications.

| Loop diuretics | furosemide, torasemide, and bumetanide |
| --- | --- |
| Other antibacterial drugs | aminoglycosides (gentamicin, amikacin, etimicin, streptomycin, tobramycin etc), amphotericin B, piperacillin-tazobactam, meropenem, imipenem-cilastatin |
| Other nephrotoxic medications | cyclosporine, tacrolimus, platinum compounds, vasopressors/inotropes (dobutamine, dopamine, epinephrine, isoproterenol, norepinephrine and vasopressin) |
